# The health effects of extreme heat

**DOI:** 10.4102/phcfm.v18i1.5372

**Published:** 2026-04-30

**Authors:** Sa’ad Lahri

**Affiliations:** 1 Division of Emergency Medicine, Department of Family and Emergency Medicine, Faculty of Medicine and Health Sciences, Stellenbosch University, Cape Town, South Africa

**Keywords:** extreme heat, heat-related illness, climate change and health, heat stroke, climate-resilient health systems

## Abstract

Extreme heat is a significant direct health threat from climate change, with rising temperatures and frequent heatwaves increasingly stressing communities and health services across Africa. High baseline temperatures, widespread outdoor labour, limited cooling access and structural vulnerabilities heighten population exposure. The physiological impacts are severe: from extreme heat overwhelming thermoregulation, leading to dehydration, cardiovascular strain, direct cellular injury and potentially rapid progression to heat exhaustion, to the most severe and dangerous form, heat stroke, which is a medical emergency characterised by a core body temperature > 40 °C and central nervous system dysfunction such as confusion, seizures or coma, leading to multiorgan dysfunction. Heat also exacerbates chronic conditions like heart failure, asthma and kidney disease. Beyond clinical presentations, community-level evidence shows heat causes sleep disturbance, irritability and significant reductions in productivity. Vulnerable groups include infants, older adults, pregnant women, individuals with chronic diseases and outdoor workers. Maternal and neonatal health is particularly at risk, with links to preterm birth, stillbirth and hypertensive disorders. Primary health care is central to addressing this threat through early recognition, prompt cooling, hydration, medication review and tailored counselling for low-resource settings and environments. A proactive integration of heat-health interventions into routine primary care is therefore critical to building climate-resilient health systems and safeguarding vulnerable populations.

## Introduction

Extreme heat is increasingly recognised as a major health threat, yet many primary care settings in Africa lack practical, synthesised guidance on its clinical and community-level impacts.^1,2^ The purpose of this article is to provide a clear and concise overview of the health effects of extreme heat that are most relevant to primary health care, and to outline the key mechanisms, vulnerabilities and clinical considerations that practitioners should be aware of.

Extreme heat is emerging as a major cause of global morbidity and mortality as temperatures rise and heatwaves become more frequent, longer and more intense.^1^ Studies of temperature-related mortality show that non-optimal temperatures contribute significantly to global deaths, with heat-related mortality increasing fastest in regions that already experience high baseline temperatures.¹ Many African countries face additional vulnerability because of widespread outdoor labour, informal housing, limited access to cooling and constrained water and energy supply systems. These environmental and socio-economic conditions heighten population exposure and reduce the capacity to adapt to extreme heat.^1,2^

Climate-related stressors already influence service delivery, patient presentations and health worker well-being across the primary care platform. A recent review examining climate change and primary health care identified heat as a key hazard affecting workload, medication safety and continuity of care in African settings.^2^ Primary care facilities therefore play a central role in recognising, managing and preventing heat-related illness.^2^

A global meta-review by Thiel et al. further highlights the breadth of conditions associated with heat exposure, including electrolyte disturbances, cardiovascular and respiratory disease, renal injury, mental health effects and increased infectious disease incidence.^3^ These findings show that heat interacts with climatic factors such as humidity and with social factors such as age, disability and socio-economic status.^2,3^ This underscores the need for primary care practitioners to understand both the physiological effects of heat and the social vulnerabilities that shape risk.

## Mechanisms of heat exposure

Human thermoregulation maintains core temperature through conduction, convection, radiation and evaporation. When environmental temperatures rise, the hypothalamus increases skin blood flow to enhance heat loss. Under significant heat stress, skin blood flow may reach 6 L to 8 L per minute.^4^ Sweating provides the dominant cooling mechanism, but high humidity greatly reduces evaporation. Once humidity exceeds roughly 75%, sweat cannot evaporate effectively, causing heat to accumulate.

When environmental heat exceeds the body’s ability to dissipate it, core temperature rises, and thermoregulatory mechanisms become overwhelmed, leading to uncompensated hyperthermia. There are five major physiological processes triggered by heat exposure that can progress to organ dysfunction: ischaemia, heat cytotoxicity, systemic inflammation, disseminated intravascular coagulation and rhabdomyolysis.^5^ These interacting mechanisms reduce blood flow to vital organs, damage cell membranes, increase permeability to toxins and pathogens and promote widespread inflammatory and coagulation disturbances. The combined effects can impair the heart, kidneys, brain, liver, pancreas, lungs and gastrointestinal tract, creating a cascade of multi-organ stress that may rapidly progress to failure if cooling and resuscitation are delayed. Underlying these pathways are cellular derangements such as mitochondrial dysfunction, oxidative injury and endotoxin translocation from the gut, which further amplify tissue damage. In severe cases, these mechanisms reinforce one another, producing a self-propagating cycle of cellular injury and circulatory collapse characteristic of heat stroke.^5^

Heat stress also impairs fluid balance and cardiovascular stability through vasodilation, dehydration and plasma volume loss, which increase cardiac workload and reduce renal perfusion.^4^ These responses disproportionately affect older adults and individuals with chronic disease. These responses disproportionately affect older adults and individuals with chronic disease, as age-related declines in sweat gland function and thirst perception reduce physiological adaptability, while medications commonly used in chronic disease management, including diuretics, anticholinergic medications, beta blockers, antipsychotics and certain antidepressants, further impair thermoregulation and exacerbate fluid loss.^4,5^

## Types of heat injury

*Mild heat-related illnesses* include heat rash, heat oedema, heat cramps and heat syncope.^4^ Heat rash presents as small pruritic vesicles caused by sweat duct blockage and local inflammation.^5^ Heat oedema results from peripheral vasodilation and dependent fluid pooling, especially in the legs.^4,5^ Heat cramps arise from painful spasms in large muscle groups because of salt loss during sweating, while heat syncope is a brief loss of consciousness with rapid recovery following peripheral vasodilation and dehydration.^5^ These conditions usually resolve with rest, cooling and rehydration.^5^

*Heat exhaustion* is a moderate heat-related illness characterised by fatigue, weakness, headache, dizziness, nausea and tachycardia.^5^ It typically follows recent exposure to high temperatures and is driven by dehydration and sodium depletion.^4,5^ Core temperature may be normal or mildly elevated. Without intervention, heat exhaustion can progress to heat stroke.^6^

*Heat stroke* is the most severe heat-related illness.^6^ It occurs when core temperature rises above 40 °C with central nervous system dysfunction such as confusion, agitation, seizures or coma.^6^ Mortality is approximately 10% and increases significantly with hypotension. There are two main forms. Exertional heat stroke affects younger, healthy individuals who generate large amounts of metabolic heat during strenuous activity.^5,6^ Classic heat stroke occurs during extreme ambient heat and humidity and primarily affects older adults, young children and those with chronic illness, disability or limited access to cooling.^6^

Several factors increase vulnerability to heat illness.

These include dehydration, certain medications, poor heat acclimatisation, impaired mobility, chronic heart or lung disease and renal impairment.^4,5^ Accurate core temperature measurement, ideally rectal, is essential because peripheral readings may underestimate severity. Urgent, aggressive cooling remains the cornerstone of management, particularly for heat stroke where delays significantly worsen outcomes.^4,5,6^

## Clinical effects of extreme heat

In many African settings, the health effects of extreme heat do not occur immediately but unfold over several days.^7^ Mortality and morbidity often peak 6 days to 12 days after exposure, with older adults, young children and people with chronic disease most affected.^7^ This lag effect has important implications for follow-up and monitoring during and after heatwaves.

Most heat-related deaths arise from worsening of pre-existing conditions rather than heat stroke itself.^5^ High temperatures increase the risk of acute cardiovascular events, renal injury, respiratory exacerbations and metabolic decompensation.^8^ Heat exposure also affects mental well-being, contributing to irritability, reduced concentration and sleep disturbance.

### Cardiovascular impacts

Heat places considerable stress on the cardiovascular system. A large systematic review showed that cardiovascular mortality increases by 2.1% for every 1 °C rise in temperature.^9^ Heatwaves increase cardiovascular mortality by nearly 12%. Hospitalisations for arrhythmias, heart failure, cardiac arrest and coronary syndromes rise markedly during hot periods.^9^ Mechanisms include plasma volume loss, increased blood viscosity, reduced coronary perfusion, higher myocardial oxygen demand and heightened arrhythmogenic potential.

### Respiratory and renal effects

High temperatures can exacerbate asthma and chronic obstructive pulmonary disease. Heat increases airway irritation, pollen exposure and particulate concentration. Renal effects include prerenal azotaemia, acute kidney injury and worsening chronic kidney disease. Heat-related kidney injury is common among outdoor workers.^10^ A systematic review included in the Thiel meta-review also demonstrated strong associations between high temperatures and acute kidney injury, particularly in labour-intensive sectors.^3^

### Maternal, foetal and neonatal health

Pregnancy increases metabolic heat production, plasma volume and cardiac output, reducing thermoregulatory reserve. A comprehensive review found increased risks of preterm birth, stillbirth, congenital anomalies, gestational diabetes and hypertensive disorders during periods of high temperature.^11^ Newborns are also vulnerable because of immature thermoregulation and dependence on caregiver behaviour.

### Infectious disease and heat exposure

Several infectious diseases show increased incidence during hot periods, including diarrhoeal disease, meningitis, malaria and cholera.^5^ High temperatures degrade water quality, accelerate microbial growth and alter vector behaviour.^7^ Heat stress also impairs gut integrity and immune function, heightening susceptibility to infection.^5,7^ In many African settings, hot periods coincide with water scarcity, increased reliance on unsafe water sources and reduced food safety, all of which amplify transmission of enteric pathogens.^12^ Heat also shortens the extrinsic incubation period of malaria parasites in mosquitoes and increases biting frequency, accelerating transmission.^12^ Outbreaks of meningococcal disease in the African meningitis belt are strongly linked to prolonged hot, dry conditions that promote nasopharyngeal carriage and mucosal vulnerability.^5,7,12^ These combined mechanisms contribute to higher primary care caseloads during heatwaves, particularly for dehydration, diarrhoeal illness and febrile presentations.^5^

### Mental health

High temperatures are associated with irritability, aggression, interpersonal violence and increased suicide risk.^4^ Studies from both high-income and African settings have also linked prolonged hot periods to higher rates of conflict and domestic violence.^5,12^ Heat impairs concentration and productivity, while sleep disruption worsens fatigue and destabilises chronic illness. Individuals with severe mental illness face additional risk because antipsychotics, antidepressants and anticholinergic medications reduce sweating and impair thermoregulation.^12,13^ Social factors such as isolation, limited mobility and inadequate housing further heighten vulnerability, making proactive monitoring and support during heatwaves essential for this group.^6,13^

## Community-level experiences and social vulnerability

Community research from Agincourt reported headaches, dizziness, sleep disturbance and reduced productivity during hot periods.^13^ Many households lacked effective cooling strategies and awareness of heat-related mortality. Extreme heat also amplifies existing health burdens at the community level, with residents noting worsening of chronic conditions such as hypertension, asthma and arthritis during very hot days.^13^ Communities frequently experience increased dehydration, heat-related fainting and disrupted sleep, which further compound stress, fatigue and reduced daily functioning.^7,13^ Socio-economic conditions, such as housing quality, access to water, vegetation cover, population density and disability status, all influence vulnerability.^14^

## Clinical implications

### Early recognition and assessment

Clinicians should maintain a high index of suspicion for heat-related illness in patients presenting with dizziness, confusion, syncope, tachycardia or dehydration during hot periods.^5^ High-risk groups, such as older adults, infants, people with chronic disease, outdoor workers and individuals on medications that impair thermoregulation (for example, diuretics, anticholinergic medications, beta blockers, antipsychotics and antidepressants), should be assessed thoroughly for dehydration, electrolyte imbalance and signs of heat stress.^4,5,6^

### Cooling and hydration

Immediate cooling is essential. Approaches include removing excess clothing, shade, airflow, spraying water on the skin and using fans.^6^ Oral rehydration is appropriate for mild to moderate dehydration, while intravenous fluids are used for severe dehydration or suspected heat stroke. Fluid resuscitation must be carefully balanced in patients with cardiac or renal disease. Antipyretics and dantrolene are not effective in the treatment of heat stroke.^5,6^

### Immersion cooling and ice baths

For suspected heat stroke, full-body cold water immersion or ice-bath cooling is the fastest method to reduce core temperature.^5^ Immersion can reduce temperature by 0.15 °C to 0.20 °C per minute.^6^ When full immersion is not possible, partial immersion or ice packs to the axillae, groin and neck provide effective alternative cooling.^5,6^ Continuous core temperature monitoring is ideal but may be limited in primary care settings.

The clinical implications for early recognition, cooling and hydration are summarised, along with signs and priority actions for key heat-related conditions, in Table 1.

**TABLE 1:** Clinical summary of heat-related illnesses: Signs, key differences and priority management actions.

Condition	Key signs	Priority actions
Exertional heat injury (typically younger, healthy adults)	Occurs during strenuous activity; can progress to exhaustion or stroke	Move to a cool, shaded area; stop activity immediately; cooling and oral rehydration
Classic heat injury (affects very young, older adults, people with chronic illness)	Occurs in high environmental heat; often affects vulnerable groups	Move to a cool environment; gentle cooling and hydration
Heat oedema and/or heat rash and/or heat cramps	Ankle or foot swelling; itchy rash; painful muscle spasms	Elevate legs; oral rehydration salts; loosen clothing, allow cooling
Heat exhaustion	Dizziness, weakness, anxiety; pale, cool, clammy skin; tachycardia, nausea, low blood pressure	Remove excess clothing; mist skin and use fans; oral rehydration if able; escalate if no improvement
Heat stroke (medical emergency)	Core temperature > 40 °C; confusion, agitation, seizures, coma; hot, flushed skin	Aggressive cooling; cold water immersion or ice-bath; ice packs to axillae, groin, neck; IV fluids; urgent hospital transfer

## Community-oriented primary care

Beyond individual patient assessment and acute management, primary health care has a central role in reducing preventable morbidity during periods of extreme heat. This includes supporting community preparedness and ensuring facility-level readiness for surges in patient demand. The following sections outline these broader responsibilities.

### Community engagement

Many communities adapt to the changing climate and increasing heat over time, for example, by having night markets. Health facilities can support community resilience by disseminating heat alerts, educating households on cooling strategies and encouraging neighbour checks for older adults and people with disabilities.^7^ Outreach efforts should prioritise informal settlements, rural communities and populations with limited access to water or electricity.^7^ Community health workers can be instrumental in these strategies, but they also need protective clothing and flexible working hours to avoid heat injury.^14^ Health promotion should also include occupational health issues on farms or other workplaces to reduce the risk of heat injury, particularly for manual labour.^14^ Some communities have suggested that heat refuges may be needed so people can escape extreme temperatures in informal housing. Increasing natural shade in urban areas through the planting of trees can also assist in reducing temperatures.^14^

### Facility readiness

Primary care facilities should develop heat action plans that outline cooling strategies for waiting areas, ensure a reliable water supply, maintain backup or alternative energy sources for fans, air conditioning or refrigeration and train staff on heat-related illness.^2,13^ Changes to infrastructure can reduce temperatures inside facilities, such as highly reflective white roof paint, insulation in the ceiling, use of natural ventilation or creation of additional shade areas. Strategies can also be implemented to reduce the number of patients waiting outside in high temperatures, such as appointment systems or better mobile primary care coverage in the catchment area. Patients may be unwilling to walk long distances to the facility during the heat of the day in rural areas, and opening hours may need to be shifted. Monitoring local temperature forecasts (early warning systems) and aligning staffing patterns with heatwave periods can help manage surges in patient volume.^2,7^ Facilities should also incorporate extreme heat into major incident planning, including clear triggers for escalation, rapid triage protocols and coordination with emergency services. Strengthening communication systems and community outreach during heatwaves can further reduce preventable morbidity.

## Conclusion

Extreme heat is an escalating health challenge across Africa. Its physiological effects, interactions with chronic illness and wider social consequences place significant demands on primary health care. Integrating heat awareness into routine practice, medication review, pregnancy care, community outreach and facility preparedness is essential for strengthening climate-resilient health systems and protecting vulnerable populations.

### Key take-home points

- Extreme heat causes exertional and classic heat injuries that affect both healthy active individuals and vulnerable groups.
- Heat stroke is the most severe outcome of extreme heat, defined by core temperature above 40 °C with neurological dysfunction.
- Rapid cooling is the key lifesaving treatment for heat stroke, and antipyretics or dantrolene are not effective.
- Mild and moderate heat-related illnesses improve with rest, cooling and hydration, but heat stroke always requires hospital admission.
- Most heat-related illnesses are preventable through hydration, shade, ventilation and focused support for high-risk groups.
